# Progress in Neurology

**Published:** 1901-07-20

**Authors:** 


### PROGRESS IN NEUROLOGY.

(*Continued from page* 233.)

**Occupation Neuroses.**—Savill [8] says that continual over-use of a certain group of muscles is apt to produce five symptoms—at first localised in one part and only affecting a certain movement, but tending ultimately to spread to other muscles and to involve other movements. In order of frequency these symptoms are as follows: (1) stiffness, cramp, or tonic muscular spasm, which is generally accompanied by (2) pain (if there be no muscular spasm there is rarely any pain); (3) weakness, which rarely amounts to actual paralysis; (4) tremor may be present; (5) in the early stages and throughout the course of a large number of cases there is no apparent alteration of nutrition, but when there is paresis it may be accompanied by atrophy, and when spasm is present hypertrophy may ensue. Paresis, if present, is accompanied by a slight degree of the reaction of degeneration. In most cases the symptoms appear in the order above given. If the spasm and pain are pronounced, the disease will probably be arrested at this stage because the patient is compelled to cease his occupation; but if they are not pronounced, paresis and perhaps obvious atrophy may supervene. The diagnosis of these conditions is as a rule easy. Peripheral neuritis, slowly oncoming hemiplegia, and disseminated sclerosis may imitate the disease, the characteristic of which is that the disability attends—at least at first—some one habitual action only. The pathology is obscure, but by analogy it is probably a lesion of the anterior cornual cells of the spinal cord. Thus in the case of the cerebral cortex it is known that an irritative lesion gives rise to spasm, a partial destruction to tremor, and a destructive lesion to paralysis. In the special case of an occupation neurosis the lesion is probably to be found in the lower neuron, by reason of the atrophy which may occur. It is not easy to see how the occupation brings about the lesion, but it is well known in general that, whilst increased function within certain limits gives rise to increase of structure, prolonged forced functioning of any nerve structure leads in course of time to atrophy. A neurotic tendency, either inherited or acquired, may predispose to the neurosis. Thus anæmia is a potent predisposing cause, and in many cases dates from an attack of influenza. The prognosis depends on the duration of the malady and on the degree of mischief. Thus atrophy is hardly ever recovered from, whilst simple spasm is more amenable to treatment. The treatment depends on what conditions are present. If it be spasm, pain, or tremor, rest and sedatives are our chief means. On attempting to resume his occupation the method of performing it must be modified. Thus, in writer's cramp, it is practically found that nearly all cases have held their pens in an improper manner. When paresis or atrophy is present, galvanism and massage are the remedies. The former is twice as efficacious if followed by massage. Our special attention should be directed to seeking and treating any concurrent or contributory cause, such as those mentioned above.

**Infantile Hysteria.**—Bézy[9] points out that hysteria in children is in some respects different from that of adults. Thus there are few, if any, circulatory manifestations in early life, whilst the respiratory system is almost equally devoid of manifestations. There are, however, two forms of paroxysmal cough met with in children which are decidedly hysterical in character—pseudo-whooping-cough and a form of cough accompanied by slight blood spitting. Symptoms connected with the digestive tract are more frequent; anorexia, vomiting, gastralgia, and erucations are not unfrequent, whilst localised peritonitis is occasionally simulated. The renal system is also prone to hysterical symptoms, the chief of which is incontinence. Conditions imitating various forms of gross lesion of the nervous system may occur. Thus meningitis, as represented by intense pain, constipation, vomiting, photophobia, and retraction, may be closely imitated by hysteria. Pseudo-epileptic seizures, contractures, and different forms of paralysis are all met with. Amblyopia and amaurosis may be unilatral or bilateral, and sometimes are associated with hemianæs-thesia most usually affecting the same side. Aphasia seems to be a somewhat rare manifestation of hysteria in children.

[8] Clin. Jour., Nov. 21, 1900. [9] Jour. de Méd., Dec. 25, 1900,

